# Combination Treatment with GSK126 and Pomalidomide Induces B-Cell Differentiation in *EZH2* Gain-of-Function Mutant Diffuse Large B-Cell Lymphoma

**DOI:** 10.3390/cancers12092541

**Published:** 2020-09-07

**Authors:** Sungryul Park, Seung-Hyun Jo, Jong-Hwan Kim, Seon-Young Kim, Jae Du Ha, Jong Yeon Hwang, Myeong Youl Lee, Jong Soon Kang, Tae-Su Han, Sung Goo Park, Sunhong Kim, Byoung Chul Park, Jeong-Hoon Kim

**Affiliations:** 1 Disease Target Structure Research Center, Korea Research Institute of Bioscience and Biotechnology (KRIBB), Daejeon 34141, Korea; psrv92@kribb.re.kr (S.P.); josh@kribb.re.kr (S.-H.J.); sgpark@kribb.re.kr (S.G.P.); 2 KRIBB School of Bioscience, Korea University of Science and Technology, Daejeon 34113, Korea; kimsy@kribb.re.kr; 3 Personalized Genomic Medicine Research Center, Korea Research Institute of Bioscience and Biotechnology, Daejeon 34141, Korea; kkjjhhk@kribb.re.kr; 4 Drug Discovery Division, Korea Research Institute of Chemical Technology, Daejeon 305-606, Korea; jdha@krict.re.kr (J.D.H.); jyhwang@krict.re.kr (J.Y.H.); 5 Medicinal Chemistry and Pharmacology, Korea University of Science and Technology, Daejeon 34113, Korea; 6 Laboratory Animal Resource Center, Korea Research Institute of Bioscience and Biotechnology (KRIBB), Daejeon 34141, Korea; myong@kribb.re.kr (M.Y.L.); kanjon@kribb.re.kr (J.S.K.); 7 Biotherapeutics Translational Research Center, Division of Biomedical Science, Korea Research Institute of Bioscience and Biotechnology (KRIBB), Daejeon 34141, Korea; tshan@kribb.re.kr; 8 Drug Discovery Center, LG Chem Ltd., Seoul 07796, Korea; skimbio@lgchem.com

**Keywords:** PRC2, EZH2, GSK126, pomalidomide, IMiD, DLBCL, synergistic effect, combination therapy

## Abstract

**Simple Summary:**

To overcome the potential threat of drug resistance or limit of potency, the combination treatment of drugs is a promising strategy. Around 22% of patients with GCB-DLBCL carry *EZH2* gain-of-function mutations and several PRC2 inhibitors are under clinical trials. Herein, we demonstrate that combination of GSK126 with pomalidomide synergistically inhibit tumor growth through inducing B-cell maturation and apoptosis in *EZH2* gain-of-function mutant DLBCL. Our study provides the molecular basis of the combination strategy of PRC2 inhibitors and IMiDs in DLBCLs harboring *EZH2* hyperactive mutation.

**Abstract:**

Enhancer of zeste 2 polycomb repressive complex 2 subunit (EZH2), the catalytic subunit of polycomb repressive complex 2 (PRC2), regulates genes involved in cell lineage and differentiation through methylating lysine 27 on histone H3 (H3K27me3). Recurrent gain-of-function mutations of *EZH2* have been identified in various cancer types, in particular, diffuse large B-cell lymphoma (DLBCL), through large-scale genome-wide association studies and *EZH2* depletion or pharmacological inhibition has been shown to exert an antiproliferative effect on cancer cells, both in vitro and in vivo. In the current study, a combination of pomalidomide and GSK126 synergistically inhibited the growth of *EZH2* gain-of-function mutant Diffuse large B-cell lymphoma (DLBCL) cells. Furthermore, this synergistic effect appeared to be dependent on cereblon (CRBN), a cellular receptor of pomalidomide, but not degradation of IKAROS family zinc finger 1 (IKZF1) or IKAROS family zinc finger 3 (IKZF3). RNA sequencing analyses revealed that co-treatment with GSK126 and pomalidomide induced specific gene sets involved in B-cell differentiation and apoptosis. Synergistic growth inhibition and B-cell differentiation were further validated in xenograft mouse models. Our collective results provide a molecular basis for the mechanisms underlying the combined therapeutic effects of PRC2 inhibitors and pomalidomide on *EZH2*-mutated DLBCL.

## 1. Introduction

Diffuse large B-cell lymphoma (DLBCL) is the most common type of lymphoid neoplasm accounting for 30–40% of adult non-Hodgkin lymphoma (NHL) worldwide [1]. Gene expression profiling has led to the identification of germinal center B-cell-like (GCB) and activated B-cell-like (ABC) subgroups of DLBCL according to cell origin [2]. Further genetic analyses have revealed recurrent alterations in EZH2, a catalytic component of polycomb repressive complex 2 (PRC2). In particular, gain-of-function mutations in the Su(var)3-9, Enhancer-of-zeste and Trithorax (SET) domain, including tyrosine 641 (Y641), account for 22% of GCB DLBCL, supporting the utility of EZH2 intervention for the treatment of this subtype [3]. The PRC2 complex constituting EZH2 together with suppressor of zeste 12 (SUZ12), Retinoblastoma (RB)-binding protein 4 (RBBP4), and embryonic ectoderm development (EED) displays intrinsic histone methyltransferase activity catalyzing lysine 27 methylation of histone H3 (H3K27) [4]. PRC2 plays essential roles in silencing of genes involved in cellular differentiation and maintenance of stem cells [5]. The hyperactive EZH2^Y646^ mutant, commonly identified in DLBCL, induces an aberrant increase in the repressive histone marker, H3K27me3, in turn, affecting the regulation of genes associated with cell differentiation and tumor suppression [6,7,8]. Several EZH2 and allosteric EED inhibitors have been shown to impede tumor growth, both in vivo and in vitro. The potent EZH2 inhibitor, EPZ005687, suppresses H3K27me3 in EZH2^Y641/A677^-mutant lymphoma in a dose-dependent manner [9]. Another EZH2 inhibitor, GSK126, is reported to significantly impair the growth of lymphoma with *EZH2* gain-of-function mutations in vivo [10]. Allosteric EED inhibitors directly interact with the H3K27me3-binding pocket of EED, thereby preventing the activation of PRC2 [11]. Moreover, EZH2 overexpression has been documented in several human cancer types, including liver [12], lung [13], bladder [14], breast [15], melanoma [15], and colorectal cancers [16]. Dysregulated EZH2 is reported to inhibit the expression of tumor suppressors, thus promoting uncontrollable growth [8].

Immunomodulatory drugs (IMiDs), including thalidomide, lenalidomide, and pomalidomide, possess antimyeloma, anti-inflammatory, and antiproliferative properties [17,18]. Once IMiDs engage the cellular receptor cereblon (CRBN), a ubiquitin E3 ligase, the Cul4-RBX1-DDB1-CRBN (CRL4^CRBN^) complex promotes the degradation of neosubstrates, such as IKAROS family zinc finger 1 (IKZF1) and IKAROS family zinc finger 3 (IKZF3) [19,20]. Recent global proteomic analyses have demonstrated that individual IMiDs additionally induce the degradation of different zinc finger proteins other than IKZF1 and IKZF3 [21]. In addition to its E3 ligase function, CRBN epigenetically regulates the Kv1.3 potassium channel required for CD4^+^ T-cell activation. CRBN binds directly adjacent to the Kv1.3 regulatory region, promoting H3K27me3 through interactions with EZH1/2. Moreover, thalidomide treatment competes out CRBN from the Kv1.3 locus, leading to a decrease in the H3K27me3 level [22]. From this point of view, it is possible that IMiDs influence EZH2 or the methylation status of H3K27 [22].

In this study, we examined the potential cooperativity between PRC2 inhibitors and pomalidomide, with a view to determine the efficacy of their combined administration as anticancer therapy. PRC2 inhibitors and pomalidomide synergistically and selectively inhibited the proliferation of *EZH2*-mutant DLBCL cells. Notably, this synergistic effect was dependent on CRBN, but not on the degradation of IKZF1 or IKZF3. RNA sequencing analyses revealed that co-treatment with GSK126 and pomalidomide induced B-cell differentiation in *EZH2*-mutant DLBCL cells. Our collective results support the utility of combination therapy involving EZH2 inhibition and pomalidomide for *EZH2*-mutated DLBCL and provide insights into the mechanisms underlying tumor suppression activity.

## 2. Results

### 2.1. Pomalidomide Enhances the Cytotoxic Effect of GSK126 in EZH2-Mutant DLBCL Cells

**Figure 1:**
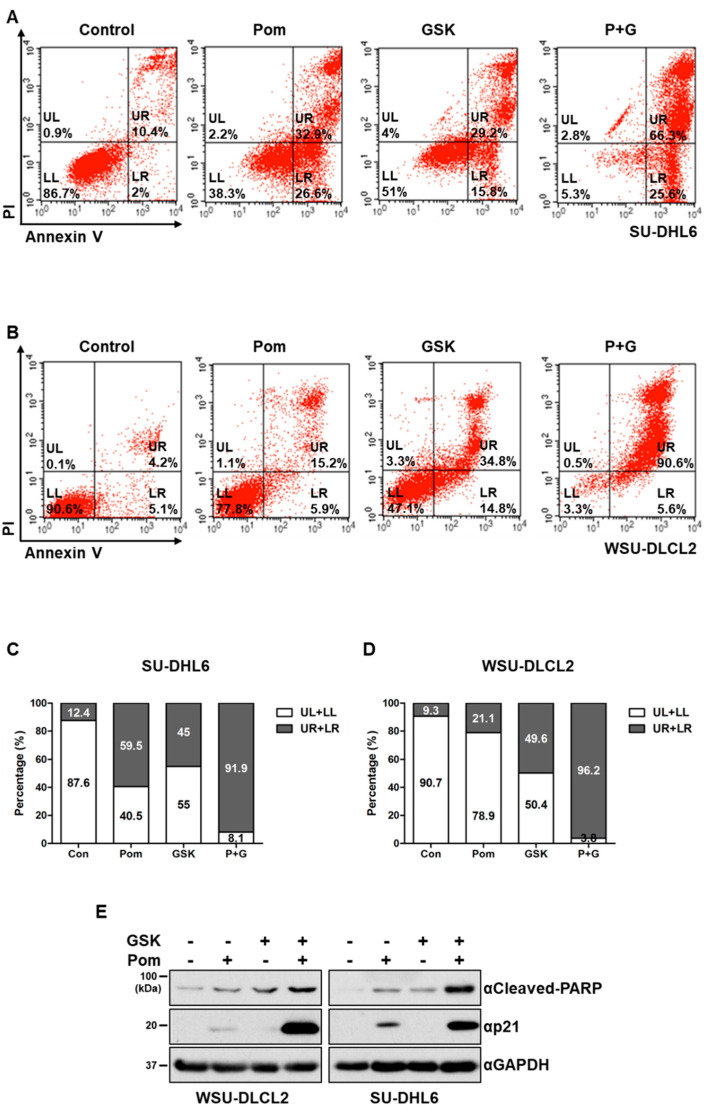

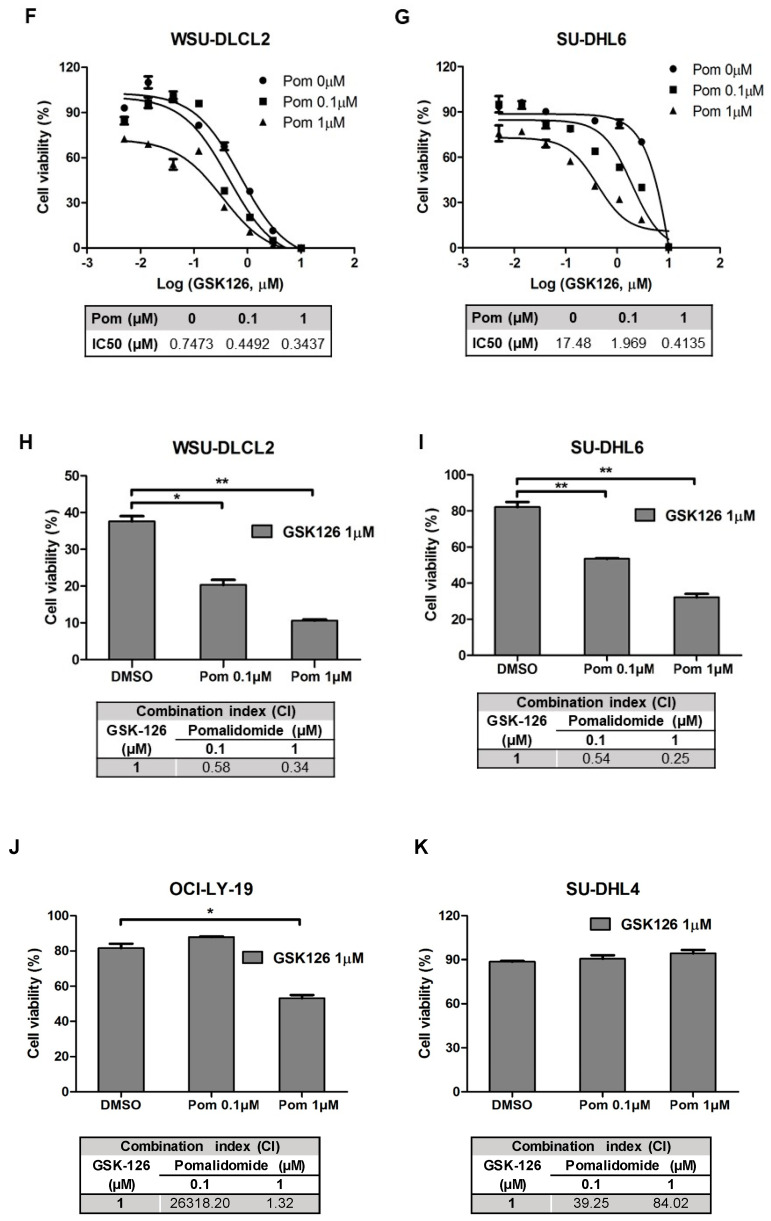
Pomalidomide enhances the cytotoxic effect of GSK126 on enhancer of zeste 2 polycomb repressive complex 2 subunit (*EZH2*)-mutant diffuse large B-cell lymphoma cells. (**A**,**B**) Annexin V-FITC (annexin V)/propidium iodide (PI) staining of SU-DHL6 and WSU-DLCL2 cells treated with dimethyl sulfoxide (DMSO, control), pomalidomide alone (Pom), GSK126 alone (GSK), or pomalidomide and GSK126 combination (P+G). LL, viable cells (annexin V-negative/PI-negative); LR, early apoptotic cells (annexin V-positive/PI-negative); UL, late necrotic cells (annexin V-negative/PI-positive); and UR, late apoptotic/necrotic cells (annexin V-positive/PI-positive). (**C**,**D**) Quantification of results from Figure 1A,B. (**E**) WSU-DLCL2 and SU-DHL6 cells were treated with DMSO, pomalidomide, GSK126, or GSK126 and pomalidomide for 48 h, and levels of cleaved PARP, p21, and Glyceraldehyde 3-phosphate dehydrogenase (GAPDH) were analyzed via western blot. (**F**,**G**) Effects of pomalidomide and GSK126 on cell viability. WSU-DLCL2 and SU-DHL6 cells were treated with the indicated concentrations of GSK126 and pomalidomide (Pom) for 6 days. (**H**–**K**) Combination index (CI) values obtained for indicated cells treated with different concentrations of pomalidomide in combination with GSK126 (1 μM). (* *p*-value < 0.05, ** *p*-value < 0.005, Student’s *t*-test).

To determine the specific impacts of pomalidomide and GSK126 on the viability of DLBCL cells harboring *EZH2* mutations, propidium iodide (PI)/annexin V staining was performed to monitor cell death. Co-treatment with pomalidomide and GSK126 induced significant SU-DHL6 (EZH2^Y641N^) and WSU-DLCL2 (EZH2^Y641F^) cell death, compared with either pomalidomide or GSK126 alone (Figure 1A–D). Consistently, cleavage of Poly (ADP-ribose) polymerase (PARP), a representative apoptotic marker, and protein levels of p21, a Cyclin-dependent kinase (CDK) inhibitor, increased in the presence of both GSK126 and pomalidomide (Figure 1E and Figure S6A). In the terminal deoxynucleotidyl transferase dUTP nick-end labeling (TUNEL) assay, co-treatment induced apoptosis in WSU-DLBCL2 cells (Figure S1A,B). To establish whether these two compounds acted synergistically, we performed a cell viability assay at a range of doses. Three EZH2^Y641^-mutant DLBCL cell lines (WSU-DLCL2, SU-DHL6, and SU-DHL10) were treated with increasing concentrations of GSK126, along with a fixed concentration of pomalidomide (0.1 or 1 μM), and cell viability was measured using the CellTiter-Glo assay. Treatment with GSK126 in conjunction with pomalidomide induced a marked decrease in viability of all three cell lines (Figure 1F,G and Figure S1C). The Chou–Talalay drug combination index (CI) was calculated to establish whether the effects of pomalidomide and GSK126 were synergistic [23]. Based on the dose-response results, CI values of the two compounds were determined as 0.58 and 0.34 in WSU-DLCL2, 0.54 and 0.25 in SU-DHL6, and 5.432 and 0.249 in SU-DHL10 in the presence of 0.1 μM and 1 μM pomalidomide, respectively (Figure 1H,I and Figure S1D). As CI values <1 signify a synergistic effect [23], we concluded that pomalidomide acted synergistically with GSK126 to exert cytotoxicity in *EZH2*-mutant DLBCL. In contrast, no synergistic effect was observed in wild-type *EZH2* DLBCL cell lines (OCI-LY-19, SU-DHL4, and Toledo) (Figure 1J,K and Figure S1E–H) and other lymphoma types, such as mantle cell lymphoma (JeKo-1, Rec-1, Mino, and Z138) and Burkitt’s lymphoma (NC-37) (Figure S1I–M). Moreover, the combination treatment did not exert additional or synergistic effects on the viability of multiple myeloma cell lines (IM-9, RPMI8226, and U266; Figure S1N–P), indicating that the synergistic antiproliferative effects of GSK126 and pomalidomide are specific for DLBCLs harboring gain-of-function mutations.

### 2.2. EED Inhibition or EZH2 Knockdown Combined with Pomalidomide Synergistically Suppresses Proliferation of EZH2-Mutant DLBCL Cells

**Figure 2:**
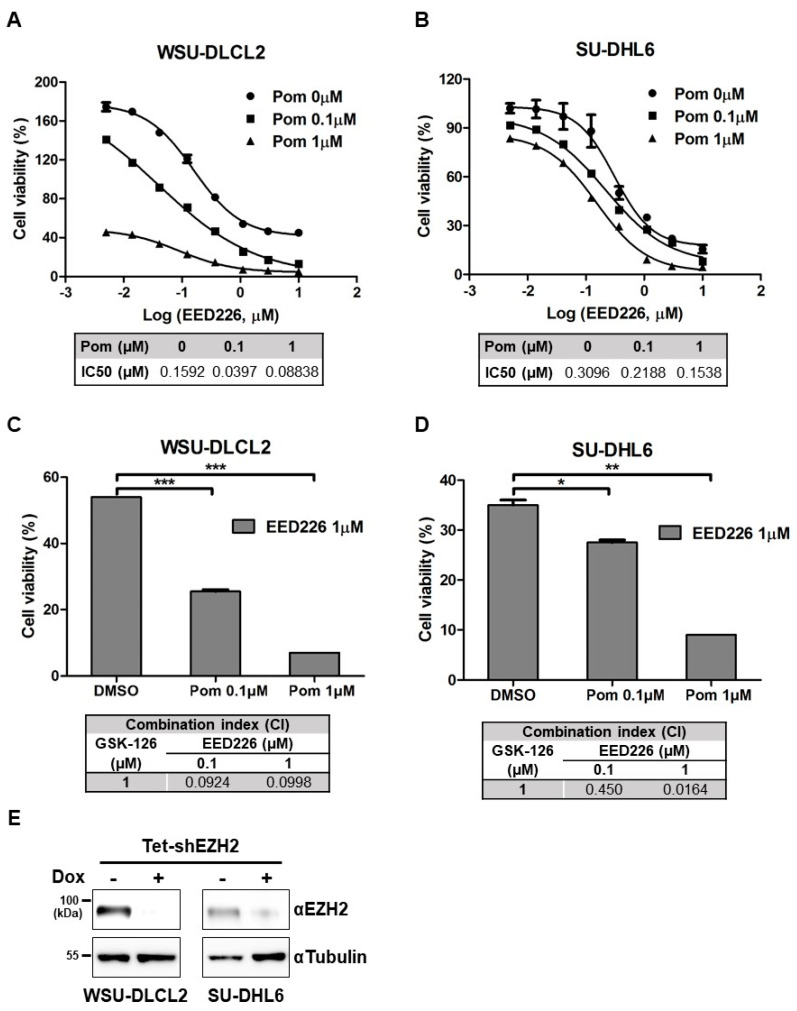

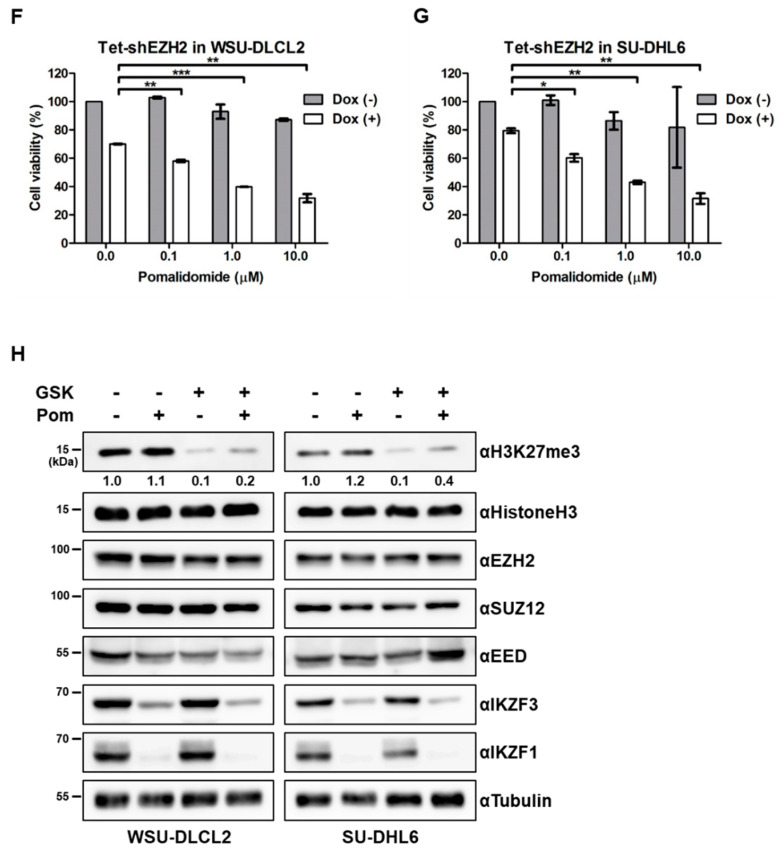
Inhibition or *EZH2* knockdown combined with pomalidomide synergistically inhibits proliferation of *EZH2*-mutant DLBCL cells. (**A**,**B**) Effects of pomalidomide and EED226 on cell viability. WSU-DLCL2 and SU-DHL6 cells were treated with the indicated concentrations of EED226 and pomalidomide (Pom) for 6 days. (**C**,**D**) Combination index (CI) values obtained for WSU-DLCL2 and SU-DHL6 cells treated with different concentrations of pomalidomide in combination with EED226 (1 μM). (**E**) EZH2 levels in Tetracycline (Tet)-inducible shEZH2 WSU-DLCL2 or SU-DHL6 cells. Cells were treated with 1 μg/mL doxycycline (Dox) for 6 days and extracts were analyzed via western blot. (**F**,**G**) Effects of *EZH2* knockdown and pomalidomide on the viability of WSU-DLCL2 and SU-DHL6 cells. (**H**) WSU-DLCL2 and SU-DHL6 cells were treated with GSK126 (GSK, 1 μM), pomalidomide (Pom, 1 μM), or both for 48 h, and cell lysates were analyzed via western blot using the indicated antibodies. (* *p*-value < 0.05, ** *p*-value < 0.005, *** *p*-value < 0.0001, Student’s *t*-test).

Given that EZH2 is a component of PRC2 and allosteric activation of EED is a prerequisite for its activity [24], we examined cellular toxicity of the EED inhibitor, EED226 [11], in combination with pomalidomide. Consistent with the data obtained using GSK126, co-treatment with EED226 and pomalidomide induced enhanced cytotoxicity against WSU-DLCL2, SU-DHL6, and SU-DHL10 cells (Figure 2A,B and Figure S2A). CI values were determined as 0.0998, 0.0164, and 0.00319 with 1 μM EED226 and pomalidomide in WSU-DLCL2, SU-DHL6, and SU-DHL10, respectively (Figure 2C,D and Figure S2B), indicating synergistic effects. We further investigated whether knockdown of *EZH2* would also exert a synergistic effect with pomalidomide. For this purpose, WSU-DLCL2 and SU-DHL6 cell lines with conditional depletion of *EZH2* were established using a Tet-inducible shRNA. In both cell lines, doxycycline treatment suppressed EZH2 expression (Figure 2E, Figures S2C and S6B). Upon knockdown of *EZH2* with doxycycline, the viability of both cell lines was decreased in a pomalidomide dose-dependent manner (Figure 2F,G and Figure S2D,E). Our collective results implied that, similar to EZH2 inhibition, *EZH2* knockdown or treatment with EED inhibitors, in combination with pomalidomide, can effectively induce synergistic antiproliferative activity.

In view of the above findings, we hypothesized that co-treatment with GSK126 and pomalidomide affected H3K27 methylation levels, thereby inducing cell death in DLBCL with EZH2 hyperactive mutations. To examine this theory, H3K27 methylation status was examined to determine EZH2 activity in WSU-DLCL2 and SU-DHL6 cells treated with GSK126, pomalidomide, or both compounds (Figure 2H and Figure S6C). As expected, GSK126 treatment induced a decrease in the H3K27Me3 level. However, co-treatment did not affect H3K27Me3 levels, compared with GSK126 alone. In addition, co-treatment did not influence the expression of PRC2 subunits (SUZ12, EED, and EZH2), suggesting that synergistic activity is not attributable to changes in histone H3 methylation or PRC2 complex integrity. Moreover, GSK126 had no effect on IKZF1/3 degradation induced by pomalidomide.

### 2.3. Synergistic Effects of GSK126 and Pomalidomide Are Dependent on CRBN, But Not on the Degradation of IKZF1 or IKZF3

**Figure 3:**
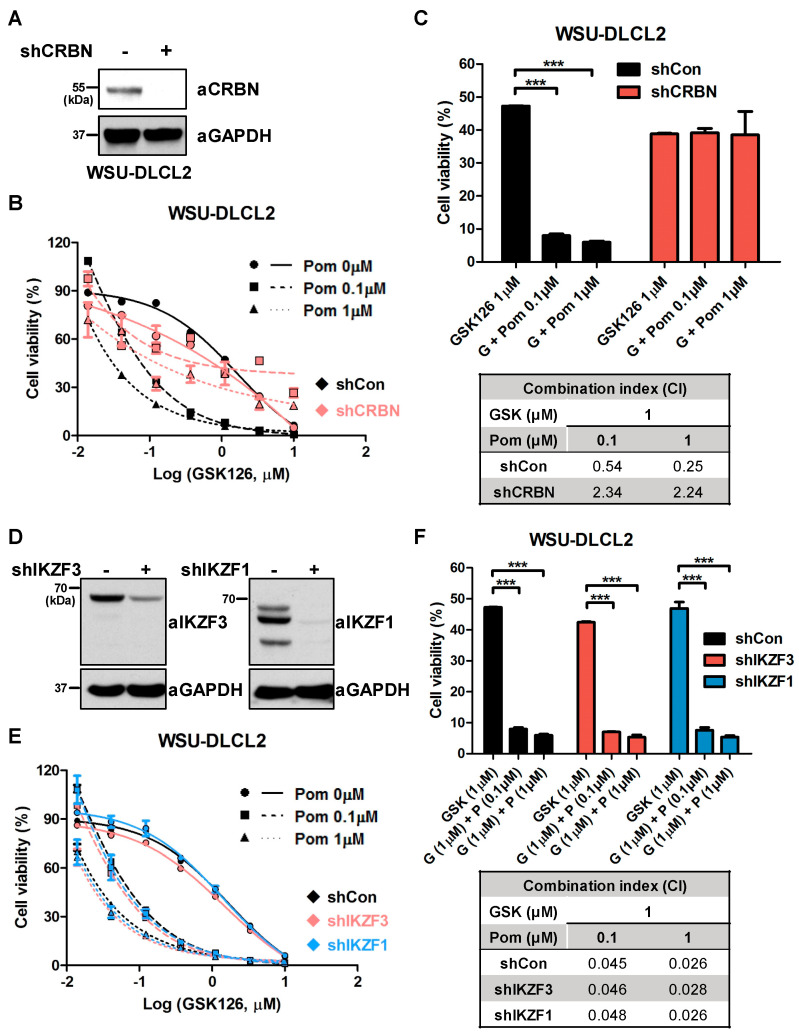
Synergistic effect of GSK126 and pomalidomide is dependent on cereblon (CRBN), but not degradation of IKAROS family zinc finger 1 (IKZF1) or 3 (IKZF3). (**A**) CRBN levels were examined via western blot in shControl and shCRBN WSU-DLCL2 cells. (**B**) Effects of pomalidomide and GSK126 on the viability of shControl and shCRBN WSU-DLCL2. Cells were treated with the indicated concentrations of GSK126 and pomalidomide (Pom) for 6 days. (**C**) Combination index (CI) values obtained for shControl and shCRBN WSU-DLCL2. Cells were treated with different concentrations of pomalidomide in combination with GSK126 (1 μM). (**D**) Western blot analysis of IKZF3 and IKZF1 levels in shControl, shIKZF1, and shIKZF3 WSU-DLCL2. (**E**) Effects of pomalidomide and GSK126 on the viability of shControl, shIKZF1, and shIKZF3 WSU-DLCL2. Cells were treated with the indicated concentrations of GSK126 and pomalidomide (Pom) for 6 days. (**F**) Combination index (CI) values obtained for shControl, shIKZF1, and shIKZF3 WSU-DLCL2. Cells were treated with different concentrations of pomalidomide in combination with GSK126 (1 μM). (*** *p*-value < 0.0001, Student’s *t*-test).

As pomalidomide belongs to the IMiD family together with thalidomide and lenalidomide, we examined whether other IMiDs, such as lenalidomide, affect proliferation activity in combination with GSK126. Similar to pomalidomide, co-treatment with lenalidomide and GSK126 synergistically inhibited WSU-DLCL2 proliferation (Figure S3A,B). IMiDs interact directly with the cellular receptor, CRBN, inducing the degradation of IKZF1 and IKZF3, thereby exerting antimyeloma activity [19]. To ascertain whether the synergistic effects of GSK126 and pomalidomide are dependent on CRBN, we generated CRBN-depleted WSU-DLCL2 cell lines with the aid of specific shRNAs (Figure 3A and Figure S6D). Compared with shControl WSU-DLCL2, pomalidomide did not enhance GSK126-induced cytotoxicity in shCRBN WSU-DLCL2 cells (Figure 3B). The CI value of shControl in the co-treatment group was <1, whereas that of shCRBN was >1, indicating that CRBN is required for the synergistic effects of GSK126 and pomalidomide on *EZH2*-mutant DLBCL (Figure 3C). We further addressed whether pomalidomide-induced degradation of IZKF1 or IKZF3 contributed to this effect. To this end, IKZF1 or IKZF3-depleted WSU-DLCL2 cells were generated using shRNA, and IKZF1 and IKZF3 levels were examined (Figure 3D and Figure S6E). As shown in Figure 3E,F, knockdown of *IKZF1* or *IKZF3* did not induce differences relative to shControl cells, suggesting that the synergistic effects of pomalidomide and GSK126 are independent of IKZF1 or IKZF3 degradation.

### 2.4. The GSK126–Pomalidomide Combination Alters Cell Plasticity and Regulates Hematopoietic Cell Lineage

**Figure 4:**
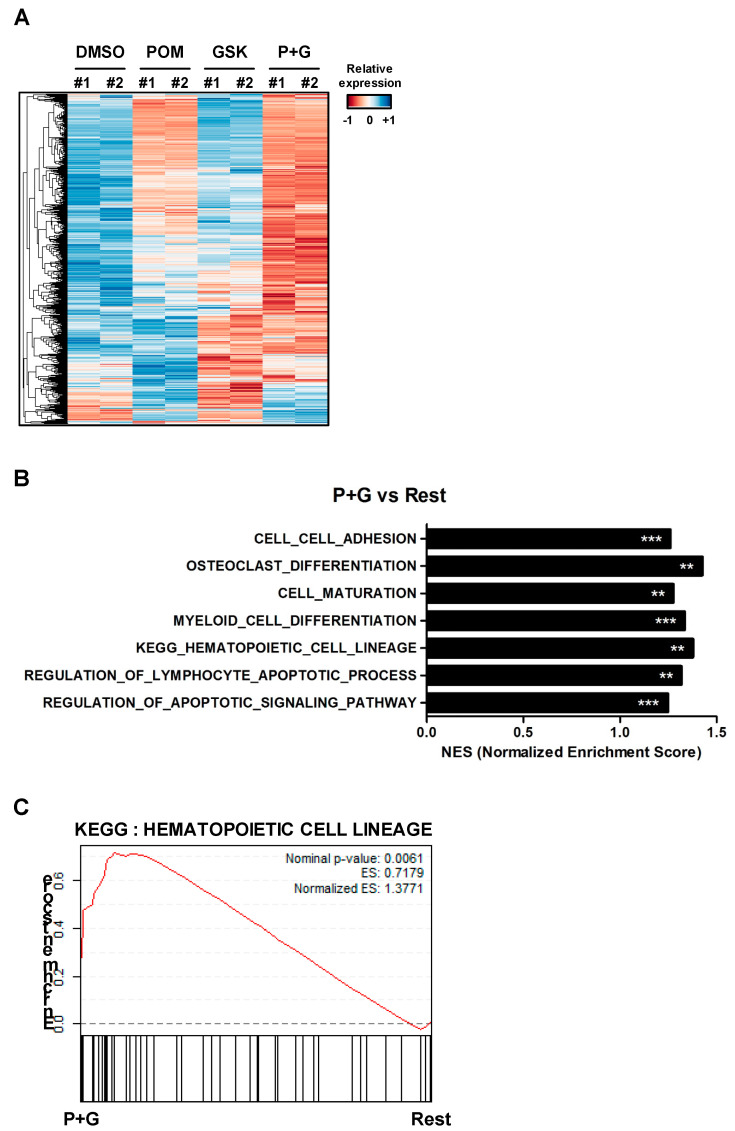

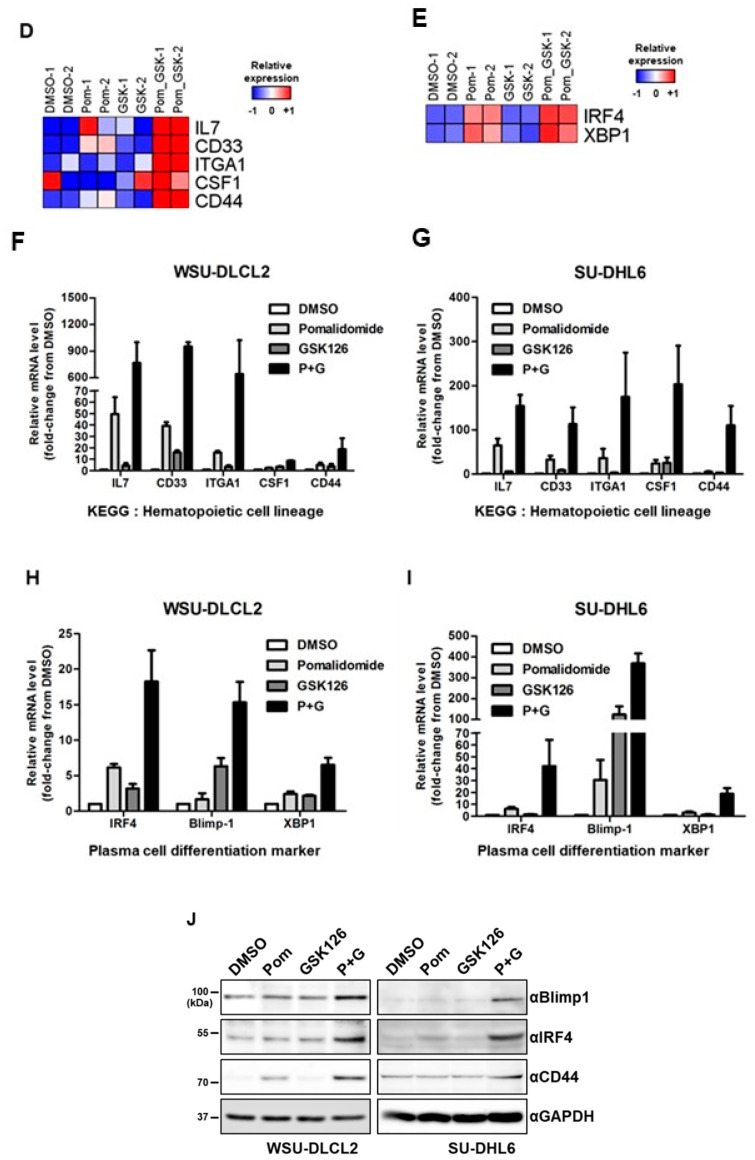
Combination treatment with GSK126 and pomalidomide changes cell plasticity and regulates hematopoietic cell lineage. (**A**) Heatmap depicting differentially expressed genes in WSU-DLCL2 treated with DMSO, pomalidomide (POM), GSK126 (GSK), or pomalidomide and GSK126 (P+G). Genes were hierarchically clustered (rows). In the heatmap, orange denotes upregulation, while blue denotes downregulation. (**B**) Gene set enrichment analysis (GSEA) of RNA-seq data. Significantly enriched gene sets in P+G relative to the remaining groups are shown as a bar chart. Asterisks indicate significance (** FDR < 0.01, and *** FDR < 0.001). (**C**) GSEA enrichment plot of the hematopoietic cell lineage pathway. (**D**) Genes involved in hematopoietic cell lineage were upregulated to a more significant extent in P+G than in other groups. (**E**) Genes essential for plasma cell differentiation were upregulated to a more significant extent in P+G than in other groups. (**F**,**G**) RT-qPCR analysis of mRNA levels of genes involved in hematopoietic cell lineage in WSU-DLCL2 and SU-DHL6 cells. (**H**,**I**) RT-qPCR analysis of mRNA levels of genes involved in plasma cell differentiation in WSU-DLCL2 and SU-DHL6. (**J**) WSU-DLCL2 and SU-DHL6 cells were treated with pomalidomide (Pom, 1 μM), GSK126 (1 μM), and P+G for 48 h. Cell lysates were analyzed via western blot using the indicated antibodies.

To analyze transcriptional changes induced by the combination of GSK126 and pomalidomide, WSU-DLCL2 cells were treated with dimethyl sulfoxide (DMSO), pomalidomide, GSK126, or a combination of GSK126 and pomalidomide for 24 h, followed by RNA sequencing analysis. Hierarchical clustering and differential expression analysis showed that the pomalidomide–GSK126 combination differentially regulated subsets of genes, compared with DMSO, pomalidomide, or GSK126 alone (Figure 4A and Figure S4A–C). Gene set enrichment analysis (GSEA) revealed enhanced expression of genes involved in cell plasticity, such as hematopoietic cell lineage (Normalized enrichment score (NES) = 1.377) and myeloid cell differentiation (NES = 1.334) as well as lymphocyte apoptotic processes (NES = 1.317) in co-treated cells (Figure 4B–E and Figure S4D,E). Consistent with our cell viability data, genes involved in apoptosis were upregulated in the co-treatment group (Figure S4F). Increased expression of genes related to hematopoietic cell lineage (interleukin 7 (IL7), CD33, integrin subunit alpha 1 (ITGA1), colony stimulating factor 1 (CSF1), and CD44) was validated in WSU-DLCL2 and SU-DHL6 cells using quantitative reverse transcription PCR (RT-qPCR) (Figure 4F,G). IL7, an essential regulator of B-cell development, upregulates cell adhesion molecules and monocyte chemoattractant protein [25,26,27]. The other three genes (*ITGA1*, *CSF1*, and *CD33*) are abundantly expressed in plasma cells [28,29,30]. The results suggested that co-treatment altered cell polarity, thereby inducing plasma cell-like differentiation of -mutant DLBCL. Accordingly, we focused on three representative genes necessary for plasma cell differentiation, specifically, interferon regulatory factor 4 (IRF4), PR/SET domain 1 (PRDM1/Blimp-1), and X-box binding protein 1 (XBP1) [31,32]. RNA sequencing analysis showed that co-treatment enhanced the expression of *IRF4* and *XBP1* (Figure 4G), consistent with RT-qPCR findings of *IRF4*, *XBP1*, and *Blimp-1* mRNA upregulation in WSU-DLCL2 and SU-DHL6 (Figure 4H,I). Additionally, we examined protein levels of Blimp-1, IRF4, and CD44 via western blot analysis. CD44, an adhesion molecule, is highly expressed in plasma cells, but barely in GCB cells [33,34,35,36]. Blimp-1 and IRF4 are essential transcriptional regulators in plasma cell differentiation. IRF4 deficiency leads to impaired expression of B-cell activation-induced deaminase and suppression of class-switch recombination [37]. Blimp-1 plays a critical role in the terminal differentiation of B-cells into Ig-secreting plasma cells [38]. The levels of all three proteins were increased in WSU-DLCL2 and SU-DHL6 cells (Figure 4J and Figure S6F), collectively indicating that combined treatment with pomalidomide and GSK126 modulates cell plasticity and promotes plasma cell differentiation in EZH2-mutant DLBCL.

### 2.5. GSK126 and Pomalidomide Synergistically Inhibit Tumor Growth In Vivo

**Figure 5:**
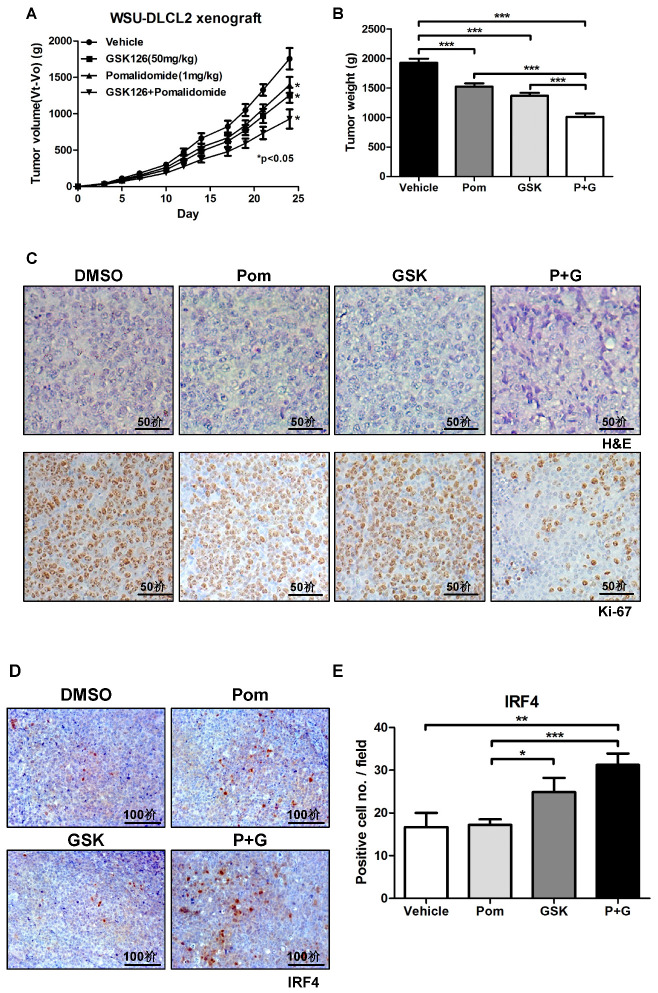
Combination of GSK126 with pomalidomide synergistically inhibit tumor growth in vivo. Specific pathogen-free CB17/SCID mice (*n* = 6) were implanted subcutaneously with WSU-DLCL2 cells (1 × 10^6^) and treated with vehicle, GSK126 (50 mg/kg), pomalidomide (1 mg/kg), or GSK126 + pomalidomide. (**A**) Tumor volumes were measured three times a week for 24 days. (**B**) Tumor weights were measured at the end of the experimental period. (**C**) Representative hematoxylin/eosin (H&E) staining (top) and Ki-67 immunostaining (bottom) of xenograft tumors (scale bar, 50 μm). (**D**) Representative photographs of IRF4 immunostaining (scale bar, 100 μm). (**E**) Mean numbers of IRF4-positive cells per field in xenograft tumor tissues are shown. (* *p*-value < 0.05, ** *p*-value < 0.005, *** *p*-value < 0.0001, Student’s *t*-test).

Given the findings from our in vitro analyses that co-treatment with pomalidomide and GSK126 promotes B-cell differentiation, thereby inhibiting the proliferation of *EZH2* mutant DLBCL, we further examined the potential synergistic effects of pomalidomide and GSK126 in vivo. To this end, DLBCL xenograft mice implanted with WSU-DLCL2 were intraperitoneally administered vehicle, GSK126, pomalidomide, or both compounds for 25 days. In this model, the combination treatment significantly inhibited tumor volume and weight, compared with individual treatments (Figure 5A,B). No significant weight loss was observed with either the combination or individual treatments (Figure S5A). Immunohistochemical staining additionally revealed reduced expression of Ki-67, a representative proliferative marker, along with sharp and spiky cell morphology in tumor samples treated with both pomalidomide and GSK126 (Figure 5C). Moreover, in line with transcriptome analyses, the combination treatment enhanced the expression of IRF4, a key transcription factor for Blimp-1-dependent plasma cell differentiation [39] (Figure 5D,E), and apoptotic markers, such as cleaved PARP, cleaved caspase 3, and p21 (Figures S5B and S6G). Our collective results indicated that GSK126 and pomalidomide act synergistically to promote plasma differentiation, thereby inhibiting the proliferation of *EZH2* gain-of-function mutant DLBCL.

## 3. Discussion

Several genome studies have revealed that GCB-DLBCL harbors oncogenic events such as t(14;18) translocation, and mutations in *EZH2*, *KMT2D*, *S1PR2*, or *GNA13* [40]. Among these, genetically altered *EZH2* has been detected in many cases. Given that the 5-year survival rate of GCB-DLBCL is ~59% with currently available immune chemotherapeutic agents, including R-CHOP, R-DHAP, R-ICE, and DA-EPOCH-R [41,42], targeting EZH2 to DLBCL with *EZH2* mutations may be more beneficial. Indeed, several clinical trials with EZH2 inhibitors are underway. A phase 1/2 study of tazemetostat, a potent inhibitor of EZH2, either as a single agent or combined with the steroid, prednisolone, is ongoing in DLBCL patients (NCT01897571). While a single EZH2 inhibitor may prove effective, determination of the efficacy of combination treatments with EZH2 inhibitors and other FDA-approved drugs is essential to overcome the potential threat of drug resistance or limitation of potency.

In this study, we demonstrated that combined treatment with GSK126 and pomalidomide or lenalidomide synergistically inhibited cell growth and induced apoptosis in *EZH2* gain-of-function mutant DLBCL. This synergistic activity was dependent on CRBN, but not on the degradation of the well-known substrates, IKZF1 and IKZF3, highlighting the possibility that proteins other than IKZF1 and IKZF3, which are degraded by pomalidomide and lenalidomide, contribute to the collective effect. Sievers and co-workers defined the human C2H2 zinc finger degrome targeted by IMiDs using whole zinc finger domain degradation reporters [43]. Another group identified multiple proteins degraded by IMiD-dependent CRL4^CRBN^ with the aid of quantitative mass spectrometry [21]. Based on these findings, we speculate that EZH2 inhibition, combined with degradation of one or more proteins targeted by pomalidomide or lenalidomide, may derepress genes related to plasma cell differentiation. On the other hand, CRBN could act as an epigenetic modulator in addition to its role as an E3 ligase receptor. Kang et al. [22] demonstrated that CRBN interacts with EZH1/2, repressing the expression of Kcna3 through direct binding to the Kcna3 locus in murine T-cells. Interestingly, thalidomide treatment blocks recruitment of CRBN to the Kcna3 locus, leading to derepression of Kcna3 transcription. In this regard, the combined activities of EZH2 inhibition with transcriptional regulation by IMiDs may lead to synergistic effects in hyperactive EZH2 DLBCL.

Gain-of-function mutations in *EZH2* have been reported in not only GCB-DLBCL, but also in other cancer types, including follicular lymphoma and melanoma [44,45]. Several missense mutations in the catalytic SET domain of EZH2 have been identified in about 7–12% of follicular lymphoma cases [6]. Functional analysis of EZH2 revealed that these mutations promote the levels of H3K27m3, resulting in suppression of specific genes, such as *TCF4*, *FOXP1*, *TCL1A*, *BIK*, *RASSF6P*, *and CDKN1* [6,7]. In addition, hyperactive EZH2 mutations are reported to control cell growth and metastasis through silencing of distinct tumor suppressors, such as *DCK*, *AMD1*, and *WDR19*, in cutaneous melanoma [46]. Further investigation of these combinational effects on different cancer types should provide a solid foundation for treating patients with hyperactive EZH2 mutations.

Besides acquisition of gain-of-function mutations, *EZH2* is overexpressed in various cancer types and its targeting has been shown to inhibit cell proliferation and metastasis [47]. *EZH2* with mutations in genes that encode subunits of SWI/SNF chromatin remodeling complexes is synthetically lethal [48,49]. Genetic alterations involving various subunits of SWI/SNF complexes have been detected in ~20% of human tumors [50]. *EZH2* knockdown and EZH2 inhibitor treatment are reported to impair proliferation and colony formation in SWI/SNF-mutant cancer cells. Our examination of the combined effects of GSK126 and pomalidomide in the SWI/SNF-mutant A549 (SMARCA4 mutant) and SKOV3 (ARID1A mutant) cell lines revealed no additional effects on cell viability (data not shown).

Our results showed the potential therapeutic opportunity for EZH2 inhibition and IMiDs in DLBCL with *EZH2* mutation; however, further studies will be needed to validate this co-treatment. Studies with patient-derived DLBCL cells or patient-derived xenograft (PDX) models will definitely help to prove the efficacy of this therapeutic modality. From a mechanistic point of view, it will also be intriguing to examine whether this combination treatment would promote the differentiation in primary immature B-cells.

## 4. Materials and Methods

### 4.1. Western Blot

Cells were lysed in RIPA buffer (150 mM NaCl, 1.0% IGEPAL^®^ CA-630, 0.5% sodium deoxycholate, 0.1% SDS, 50 mM Tris-HCl, pH 8.0). Lysates were separated via SDS-PAGE and transferred to a nitrocellulose (NC) membrane (GE Healthcare, Chicago, IL, USA). Blots were probed with the indicated primary antibodies, followed by Horseradish peroxidase (HRP)-conjugated anti-rabbit or anti-mouse IgG antibody, and detection was performed with a chemiluminescent HRP substrate (Thermo, Waltham, MA, USA). Antibodies for western blots are listed in Table S1.

### 4.2. Cell Cultures and Stable Cell Lines

The lymphoma cell lines, Toledo (ATCC, Cat. No. CRL-2631, RRID: CVCL_3611), SU-DHL6 (ATCC, Cat. No. CRL-2959, RRID: CVCL_2206), and SU-DHL4 (ATCC, Cat. No. CRL-2957, RRID: CVCL_0539), were obtained from American Type Culture Collection (ATCC, Manassas, VA, USA). WSU-DLCL2 (DSMZ, Cat. No. ACC-575, RRID: CVCL_1902), SU-DHL10 (DSMZ, Cat. No. ACC-576, RRID: CVCL_1889), and OCI-LY19 (DSMZ, Cat. No. ACC-528, RRID: CVCL_1878) were obtained from Deutsche Sammlung von Mikroorganismen und Zellkulturen (Braunschweig, Germany). It was confirmed that there was no mycoplasma contamination in the cells upon thawing by using the Universal Mycoplasma Detection Kit (ATCC, Cat. No. 30-1012K). All experiments were performed between passages 6 and 20 upon purchase. Each cell line was cultured in RPMI 1640 (GIBCO, Waltham, MA, USA, Cat# 11875-093) supplemented with 10% fetal bovine serum (FBS) (GIBCO, Cat. No. 16000-044) and 100x Antibiotic-Antimycotic (GIBCO, Cat. No. 15240062) diluted to 1×. Cells were maintained in a humidified incubator at 37 °C and 5% (*v*/*v*) CO_2_. Short hairpin (shRNA) knockdown cell lines were generated using the retroviral infection method. For production of lentiviral particles, HEK293T (ATCC, Cat. No. CRL-3216, RRID: CVCL_0063) cells were transfected with a lentiviral plasmid containing each gene, psPAX2, and pMD2.G at a ratio of 1:0.75:0.25. After 72 h of transfection, supernatant fractions containing lentiviruses were collected and filtered through a 0.45 μm sterile filter to remove cell debris. Cells were treated with the viral supernatant and 8 μg/mL polybrene (Sigma Aldrich, St. Louis, MO, USA, Cat. No. H9268), followed by incubation for 48 h. After viral transduction, cells were subjected to treatments and selected with 1 μg/mL puromycin. Mission shRNA plasmids for *CRBN*, *IKZF1*, and *IKZF3* were purchased from Sigma-Aldrich. An inducible shRNA Tet-pLKO-puro vector was obtained from Addgene (Watertown, MA, USA) (plasmid #21915), and *EZH2* targeting sequences were cloned as reported previously [51]. Each target sequence is listed in Table S2.

### 4.3. Compounds

Pomalidomide (Cat. No. S1567) and GSK126 (Cat. No. S7061) were purchased from Selleck Chemicals (Houston, TX, USA). EED226 was synthesized as described previously [11]. Chemicals were diluted in dimethyl sulfoxide (DMSO) and stored at −20 °C until use.

### 4.4. Cell Viability Assay

Cells were cultured in 96-well white opaque plates at a density of 1 × 10^4^/mL in the appropriate medium with FBS. After treatment with the appropriate compounds, assay plates were incubated for 6 days at 37 °C and 5% CO_2_ under >90% relative humidity. The number of viable cells was determined using the CellTiter-Glo Luminescent Cell Viability Assay (Promega Corp, Madison, WI, USA) based on quantification of cellular ATP as a marker of metabolically active cells using Victor X3 (Perkin Elmer, Waltham, MA, USA). Data were expressed as the mean percentage of duplicate cells and normalized to DMSO. Results from the cell viability assay were analyzed using the CompuSyn 1.0 software (ComboSyn Inc., Paramus, NJ, USA) developed by Chou based on the median-effect principle to determine whether the combined effects of drugs were synergistic, antagonistic, or additive. An antagonistic effect is above the slope line (combination index (CI) > 1), a synergistic effect is under the line (CI < 1), and an additive effect is on the line (CI = 1) [23].

### 4.5. RNA Sequencing and Data Processing

The RNA sequencing library was prepared using the TruSeq RNA Sample Prep Kit (Illumina, San Diego, CA, USA) and sequencing was performed using the Illumina HiSeq2000 platform to generate 100 bp paired-end reads. The human reference genome was obtained from the NCBI genome and genome indexing was performed using STAR (v.2.5.1) [52]. Sequenced reads were mapped to human genome (hg19) STAR, and gene expression levels were quantified with the count module. The edgeR (v.3.12.1) [53] package was applied to select differentially expressed genes from RNA-seq count data between DMSO, POM, GSK, and POM+GSK samples (fold change > 1.5, *p*-value < 0.05). The TMM (trimmed mean of M-value normalization)-normalized CPM (counts per million) value of each gene was added to 1 and log_2_-transformed for further analysis. A heatmap was generated using MultiExperiment Viewer (MeV) [54] and R (v.3.5.0) heatmap package (v.1.0.12). Next generation sequencing (NGS) data were deposited in the NCBI Gene Expression Omnibus under accession number GSE147320. Raw sequence tags were deposited in the NCBI Short Read Archive (SRA) under accession number SRP253527.

For GSEA analysis of RNA-seq data, we used GSEA software [55] from the Broad Institute website (v.4.0.0). Our expression dataset was analyzed against a hallmark Kyoto Encyclopedia of Genes and Genomes (KEGG) pathway and Gene Ontology (GO) gene sets (H, C2, and C5.gmt files from MSigDB v.7.0). Statistical significance (nominal *p*-value) of the enrichment score (ES) was calculated by running 500 gene set permutations. The normalized enrichment score (NES) accounted for the size of the gene set.

### 4.6. Quantitative Real-Time PCR

Total RNA was extracted using a Qiagen RNeasy kit (Qiagen, Hilden, Germany, Cat. No. 74104) and cDNA was synthesized using the RevertAid First Strand cDNA Synthesis Kit (Thermo Scientific, Cat. No. K1622). Quantitative PCR (qPCR) was conducted using the Solg™ Real-Time PCR Kit, with EvaGreen™ intercalating dye detection (Solgent, Daejeon, Korea, Cat. No. SRH91-R25h). HPRT1 was employed as the internal control. The primers used are listed in Table S3.

### 4.7. Flow Cytometry and Annexin V Staining

Apoptosis was assessed using the FITC Annexin V Apoptosis Detection Kit (BD Biosciences, Cat. No.556547) according to the manufacturer’s instructions. SU-DHL6 cells were seeded in a 150 mm cell culture dish at a density of 4 × 10^5^/mL cells per plate and treated for 6 days. Harvested cells were resuspended in annexin-binding buffer according to the manufacturer’s protocol. Next, cells were labeled using annexin V-FITC and PI. After incubation for 15 minutes at room temperature in the dark, samples were analyzed on a flow cytometer (BD Biosciences, Franklin Lakes, NJ, USA, FACSCalibur) for the detection of annexin V- and PI-positive subpopulations. DMSO-treated cells were used as the control group.

### 4.8. Immunohistochemistry

Xenograft tumor tissues were fixed in 10% neutral buffered formalin and processed to construct paraffin blocks following standard protocols [56]. Tissues were sliced into 4 μm thick sections and stained with hematoxylin/eosin (H&E). For immunohistochemical analysis, sections were blocked using 5% goat serum and incubated with antibodies against Ki-67 and IRF4. The mean number of IRF4-positive cells per field was calculated. Antibodies are listed in Table S1.

### 4.9. Xenograft Mouse Experiments

Female CB17/SCID mice (CB17/Icr-*Prkdc^scid^*/CrlCrlj, 5 weeks old) were purchased from Charles River Japan (Yokohama, Japan) and housed under specific pathogen-free conditions. Rooms were maintained under a 12 h light–dark cycle at 21 ± 2 °C. Animals were allowed to acclimatize to the local environment for one week before use in experiments. All animal experiments were approved by the Institutional Animal Care and Use Committee of Korea Research Institute of Bioscience and Biotechnology. WSU-DLCL2 cell suspensions (3 × 10^6^ cells) were injected subcutaneously into CB17/SCID mice. When tumor volumes reached ~50 mm^3^, mice were randomly divided into four groups. GSK126 and pomalidomide were dissolved in 20% Captisol^®^ and saline, respectively. Vehicle, GSK126 (50 mg/kg), and pomalidomide (1 mg/kg) were administered intraperitoneally (5 days a week for 3 weeks). Tumor volumes were measured three times a week using Vernier calipers and calculated using the formula: length (mm) × width (mm) × height (mm)/2. On day 24, tumors were surgically removed, weighed, photographed, divided into two sections, and stored in formalin and liquid nitrogen for further analysis. Body weights of mice were measured three times a week throughout the experimental period. The ethical code number for animal experiments was KRIBB-AEC-19024.

### 4.10. Statistical Analysis

One-way ANOVA, followed by Dunnett’s multiple comparison test, and two-way ANOVA, followed by Bonferroni multiple comparison test, were applied for statistical analysis using the GraphPad Prism5 software (GraphPad Software Inc., La Jolla, CA, USA). Cell viability and tumor volume data were analyzed using an independent Student’s *t*-test and significance was indicated (* *p*-value < 0.05, ** *p*-value < 0.005, *** *p*-value < 0.0001).

## 5. Conclusions

In conclusion, data from our study collectively demonstrated the synergistic effects of PRC2 inhibition and IMiDs for the first time and shed light on the molecular mechanisms by which the combination therapy induced plasma cell differentiation and eventually apoptosis in *EZH2* gain-of-function mutant DLBCL.

## Supplementary Materials

The following are available online at https://www.mdpi.com/2072-6694/12/9/2541/s1, Figure S1: Cytotoxic effect on various cancer cell lines, Figure S2: Inhibition or *EZH2* knockdown combined with pomalidomide synergistically inhibits proliferation of *EZH2*-mutant DLBCL cells, Figure S3: Cytotoxic effect of GSK126 with lenalidomide in WSU-DLCL2 cells, Figure S4: Whole transcriptomic comparative analysis between POM+GSK and rest, Figure S5: Specific pathogen-free CB17/SCID mice (*n* = 6) were implanted subcutaneously with WSU-DLCL2 cells, Figure S6: Original images of westernblot, Table S1: Antibodies used for detecting proteins, Table S2: shRNA targeting sequences for knockdown cell lines, and Table S3: Primer sequences for RT-qPCR.
