# Supplementary material for: Combination Treatment with GSK126 and Pomalidomide Induces B-Cell Differentiation in EZH2 Gain-of-Function Mutant Diffuse Large B-Cell Lymphoma
Source: Cancers (Basel). 2020 Sep 7;12(9):2541. doi: 10.3390/cancers12092541 (PMC7565736; doi:10.3390/cancers12092541)
Supplement: Supplementary file 1 [file cancers-12-02541-s001.pdf]

Supplementary Materials:

Combination Treatment with GSK126 And Pomalidomide Induces B-Cell Differentiation in EZH2 Gain-of-Function Mutant Diffuse Large B-Cell Lymphoma

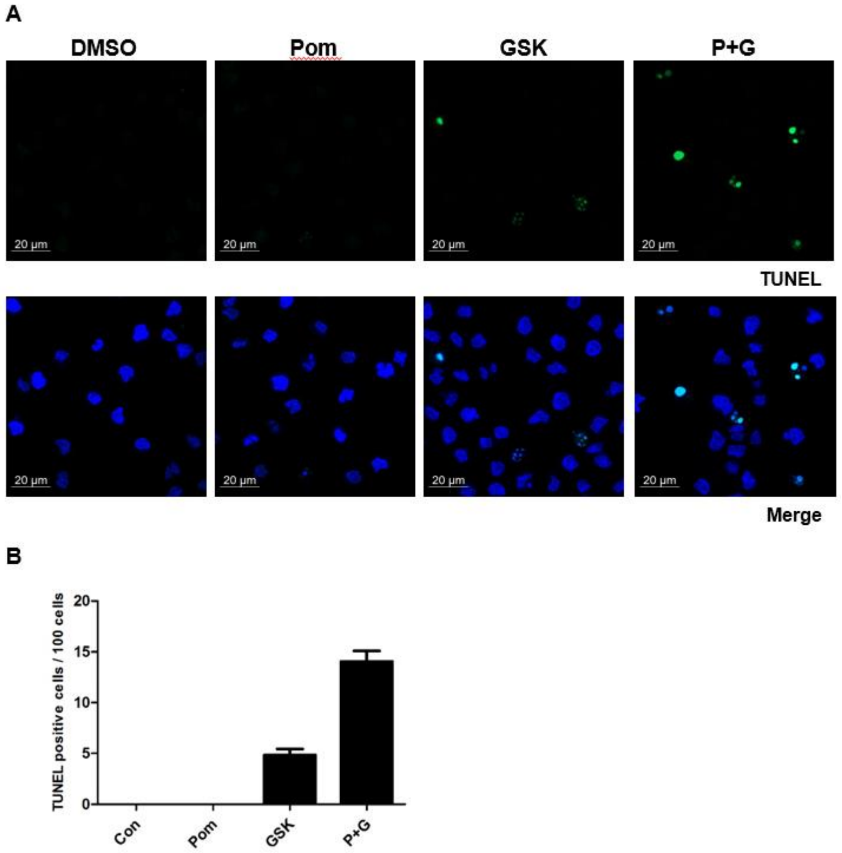

C

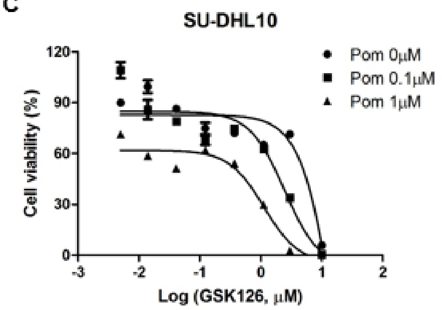

D

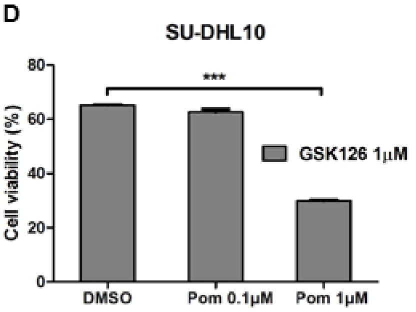

Combination index (CI)		
GSK-126 (μM)	Pomalidomide (μM)	
	0.1	1
1	5.432	0.249

E

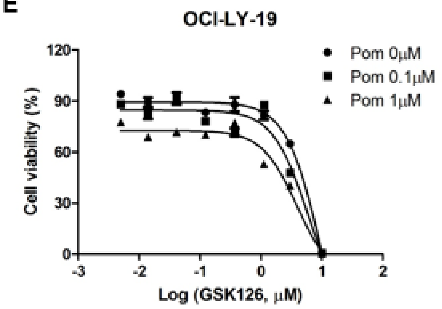

F

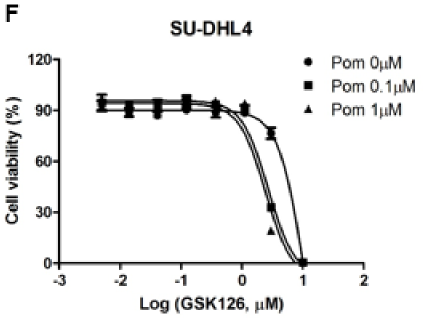

G

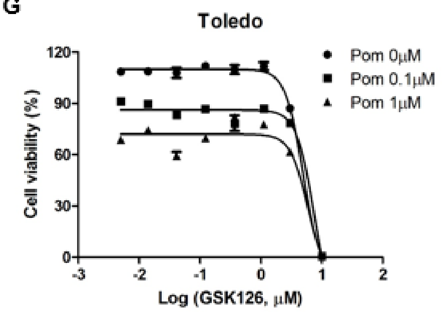

H

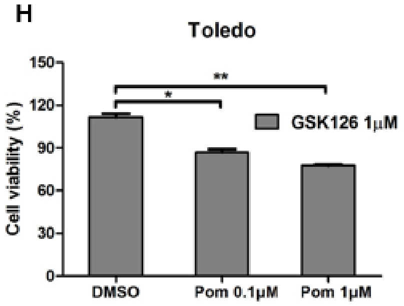

Combination index (CI)		
GSK-126 (μM)	Pomalidomide (μM)	
	0.1	1
1	17.21	5.49

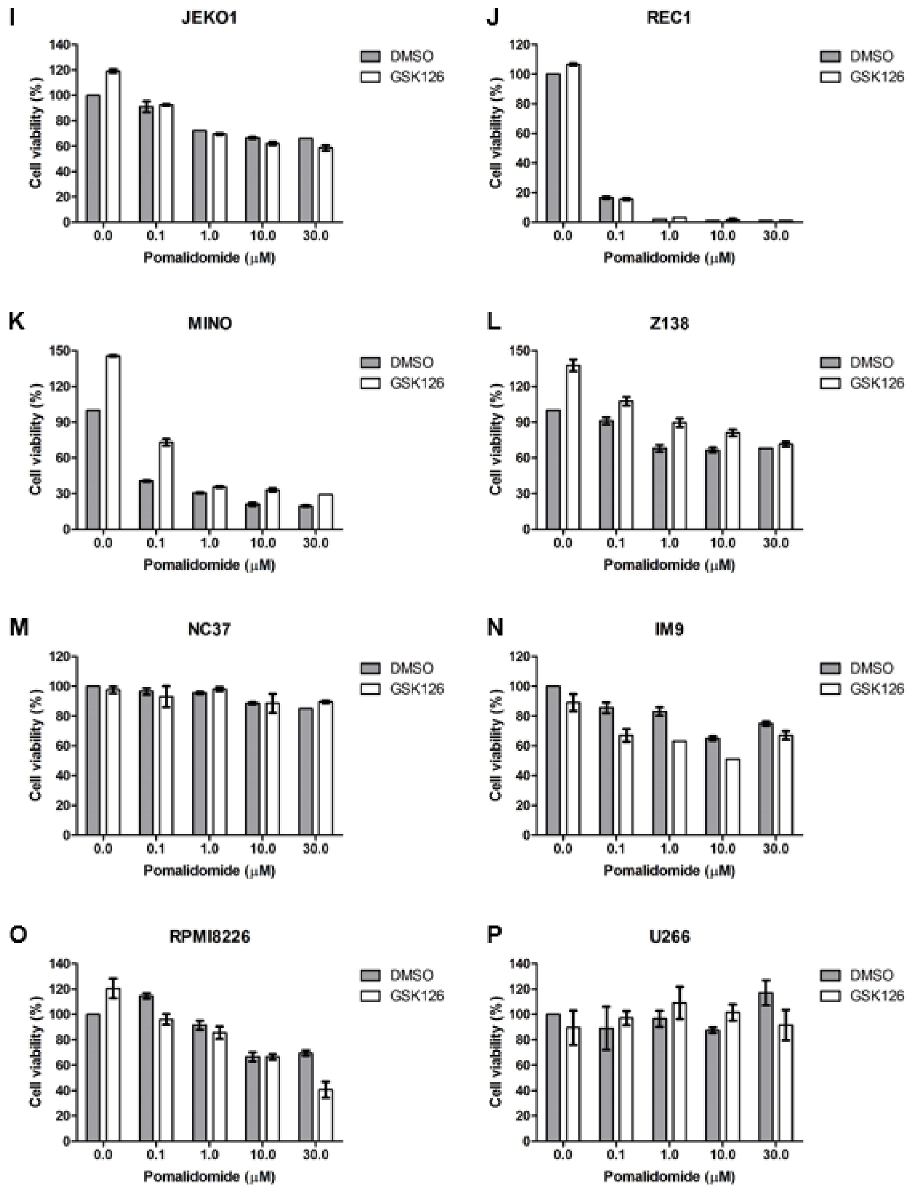

Figure S1. Cytotoxic effect on various cancer cell lines (**A**,**B**) Detection and quantification of DNA fragmentation in apoptotic WSU-DLCL2 cells by TUNEL assay after 48 hr treatment of GSK126 and pomalidomide. (**C**–**H**) Effect of GSK126 and pomalidomide combination treatment on the viability of SU-DHL10, OCI-LY-19, SU-DHL4 and Toledo in cell culture on day 6. Combination index (CI) was calculated by CompuSyn software. (**I**–**P**) Cell viability assay of EZH2 inhibitor GSK126 and pomalidomide combination treatment in myeloma cell line IM9, RPMI8226, U266 (**I**–**K**), mantle cell lymphoma JEKO1, REC1, Mino, Z138 (**L**–**O**), and burkitt's lymphoma NC37 (**P**) cell lines on day 6 (* *p*-value < 0.05, ** *p*-value < 0.005, *** *p*-value < 0.0001, student *t*-test).

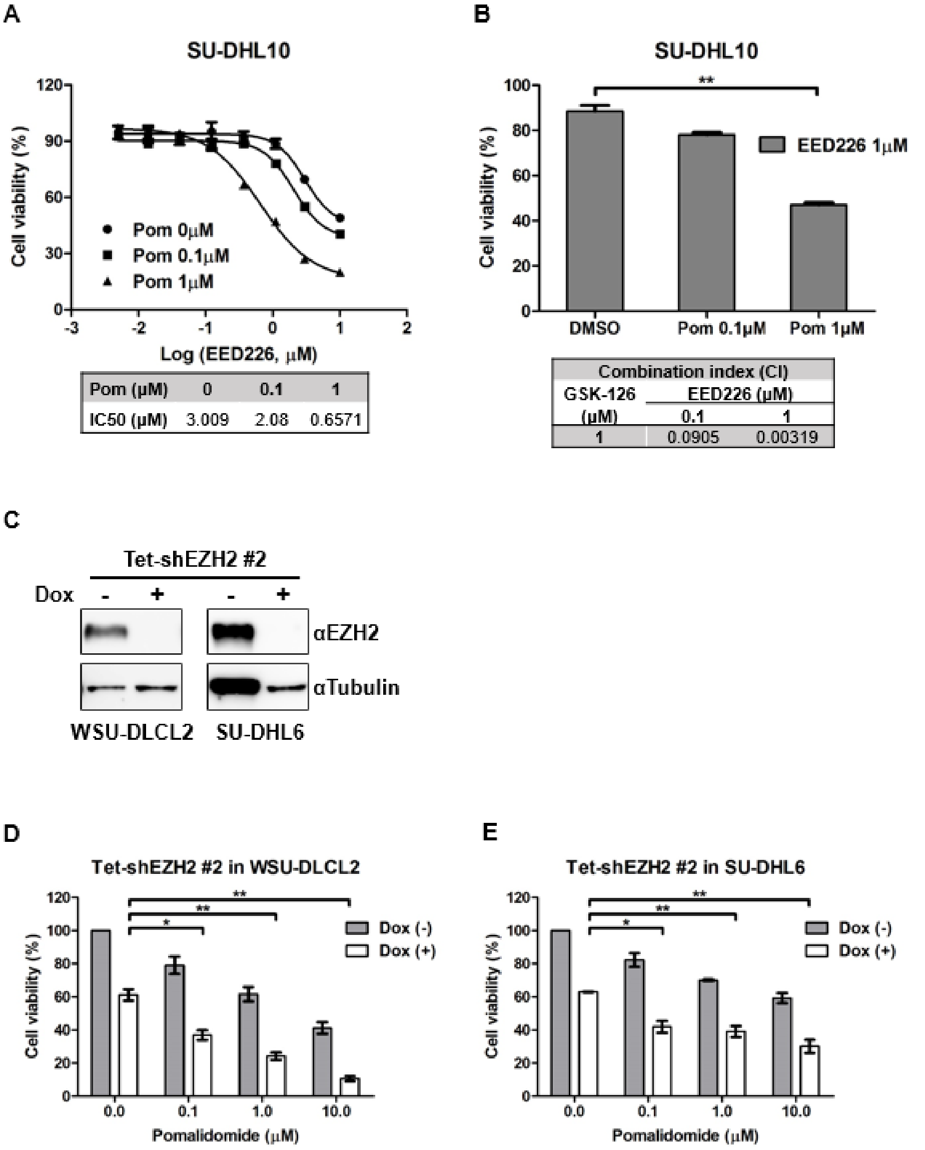

Figure S2. Inhibition or *EZH2* knockdown combined with pomalidomide synergistically inhibits proliferation of *EZH2*-mutant DLBCL cells (**A**) Effects of pomalidomide and EED226 on cell viability. SU-DHL10 cells were treated with the indicated concentrations of EED226 and pomalidomide (Pom) for 6 days. (**B**) Combination Index (CI) values obtained for SU-DHL10 cells treated with different concentrations of pomalidomide in combination with EED226 (1 μM). (**C**) Inducible shEZH2 targeting different region (#2) stable cell lines were generated in WSU-DLCL2 and SU-DHL6. Western blot validated *EZH2* knockdown after 1 μg/ml of doxycycline (Dox) treatement for 6days. *α* tubulin was used as a loading control. (**D,E**) Effect of *EZH2* knockdown and pomalidomide simultaneously on the viability. WSU-DLCL2 were treated pomalidomide as indicated concentrations and Doxycycline (Dox)- positive (1 μg/ml) or negative (water) for 6 days (* *p*-value < 0.05, ** *p*-value < 0.005,*** *p*-value < 0.0001, student *t*-test).

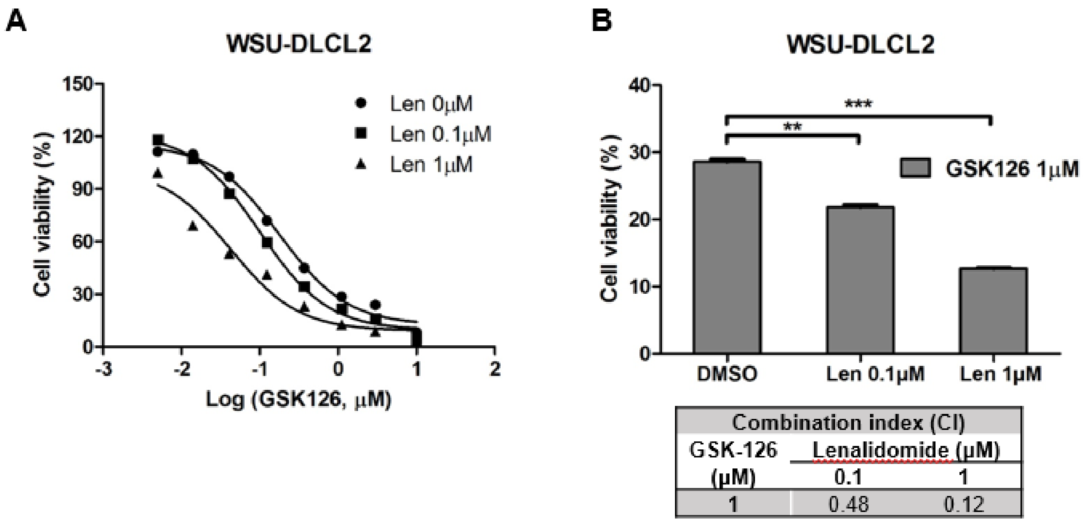

Combination index (CI)		
GSK-126 (μM)	Lenalidomide (μM)	
	0.1	1
1	0.48	0.12

Figure S3. Effect of GSK126 with lenalidomide in WSU-DLCL2 cells. (**A**) Effects of lenalidomide and GSK126 on cell viability. WSU-DLCL2 was treated with the indicated concentrations of GSK126 and lenalidomide (Len) for 6 days. (**B**) Combination Index (CI) values obtained in WSU-DLCL2 treated with different concentrations of the lenalidomide in combination with the GSK126 (1 uM) (** *p*-value < 0.005, *** *p*-value < 0.0001, student t-test).

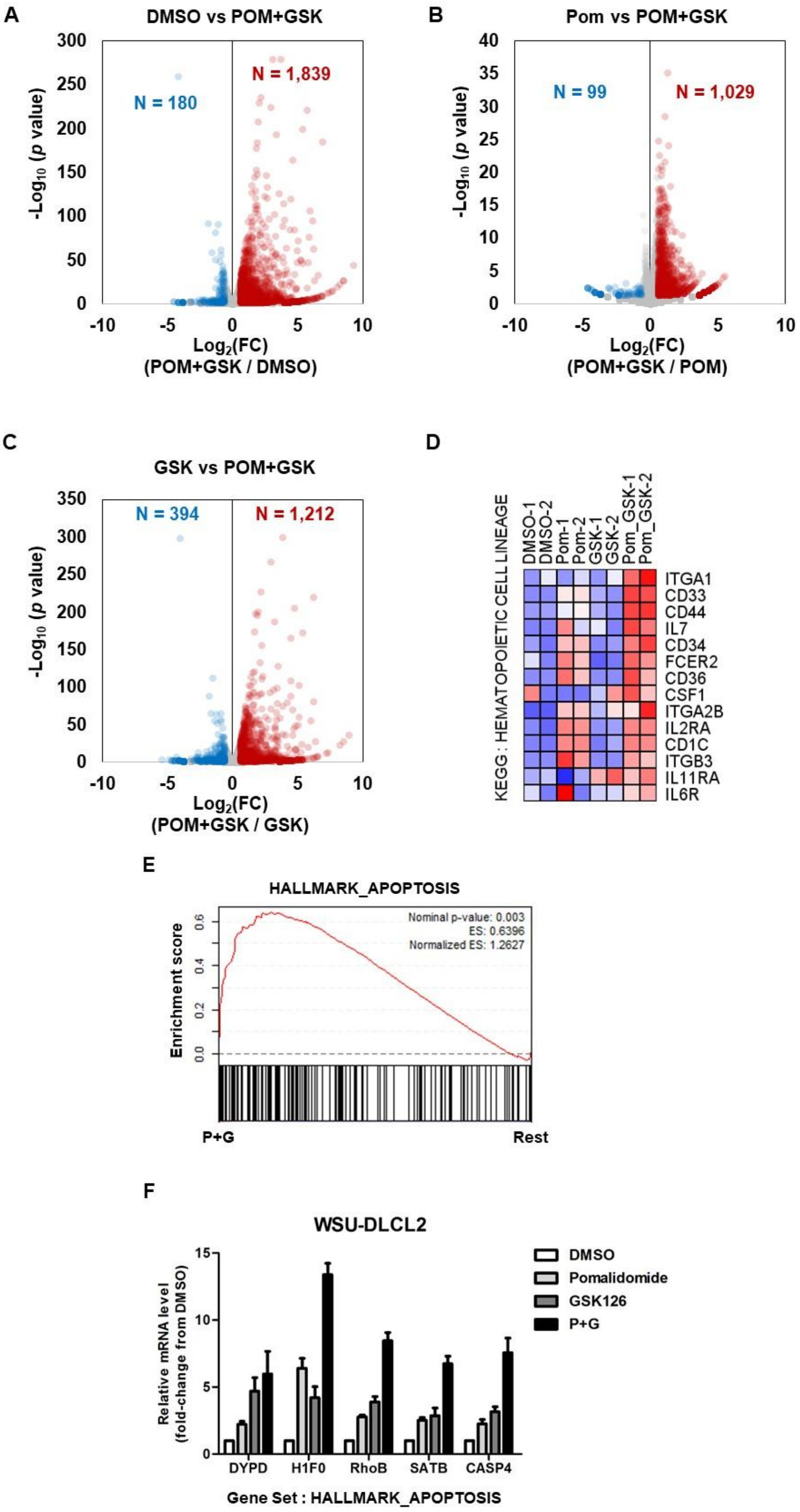

Figure S4. Whole transcriptomic comparative analysis between POM+GSK and rest. (**A–C**) Volcano plot showing the comparison of the whole transcriptomes of between POM+GSK and rest. (**A**) POM+GSK vs DMSO. (**B**) POM+GSK vs POM. (**C**) POM+GSK vs GSK. Reds: up-regulated genes; Blues: down-regulated genes. (**D**) Heatmap of Hematopoetic cell lineage gene sets. (**E**) GSEA enrichment plot of apoptosis pathway. (**F**) mRNA levels of genes related to apoptosis in GSEA were measured in WSU-DLCL2. Data are shown as mean ± standard error of mean (SEM).

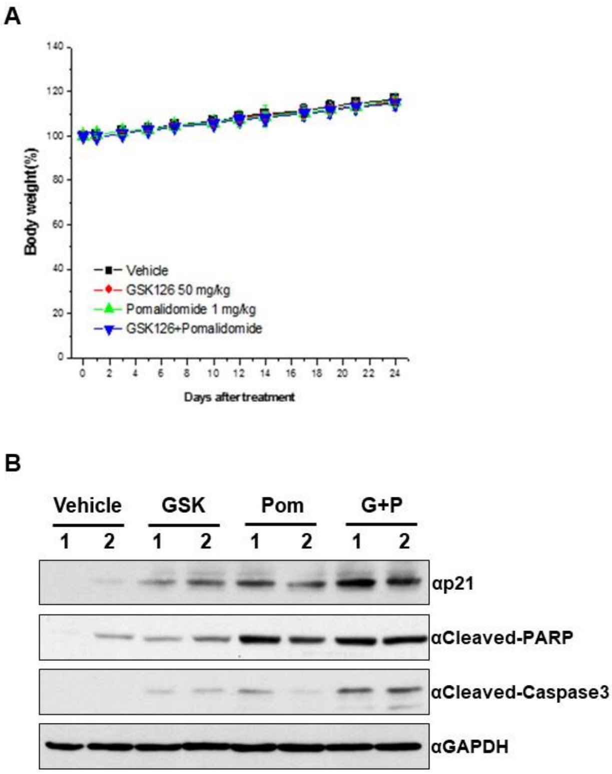

Figure S5. Specific pathogen-free CB17/SCID mice (*n* = 6) were implanted subcutaneously with WSU-DLCL2 cells (1 × 10^6 cells) and treated with vehicle, GSK126 (50 mg/kg), pomalidomide (1 mg/kg) and GSK126 + pomalidomide. (**A**) Body weights were measured three times a week for 24 days. (**B**) Immunoblot of apoptotic markers of each two representative tumor tissues sample of mouse treated GSK126 and pomalidomide, each alone and vehicle. GAPDH was loading control.

A

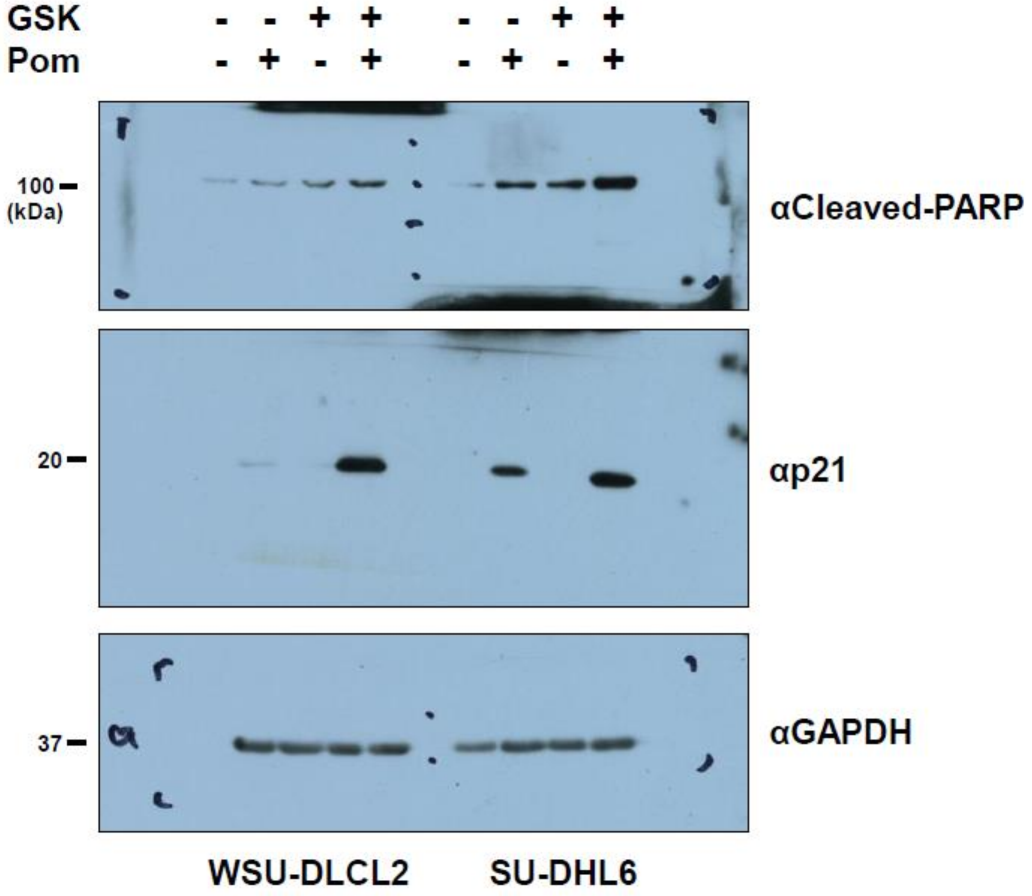
GSK - - + + - - + +
Pom - + - + - + - +
100
(kDa)
αCleaved-PARP
20
αp21
37
αGAPDH
WSU-DLCL2
SU-DHL6

B

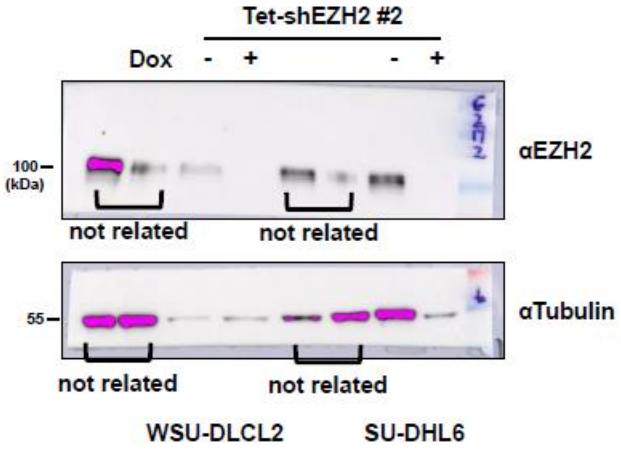
Tet-shEZH2 #2
Dox - + - +
100
(kDa)
αEZH2
not related
not related
55
αTubulin
not related
not related
WSU-DLCL2
SU-DHL6

C

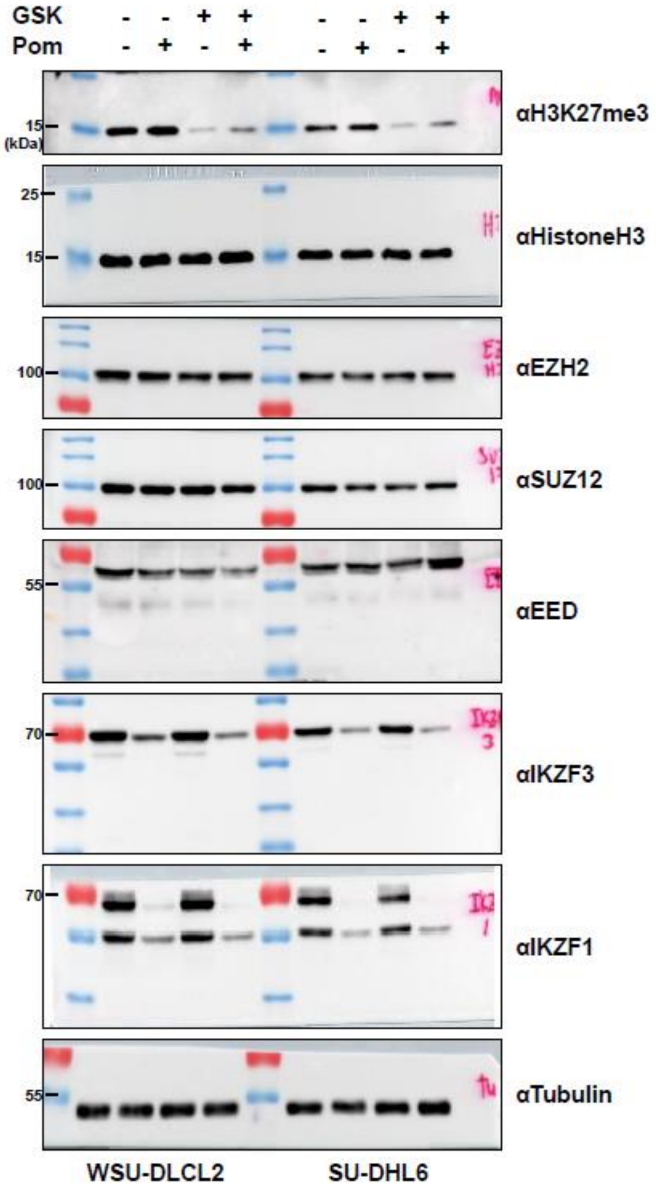
GSK - - + + - - + +
Pom - + - + - + - +
15
(kDa)
αH3K27me3
25
15
αHistoneH3
100
αEZH2
100
αSUZ12
55
αEED
70
αIKZF3
70
αIKZF1
55
αTubulin
WSU-DLCL2
SU-DHL6

D

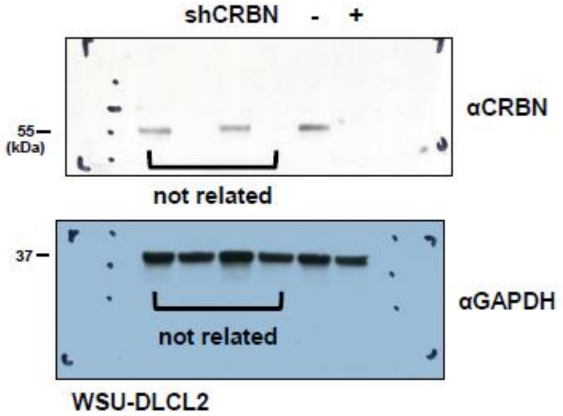
shCRBN - +
55
(kDa)
αCRBN
not related
37
αGAPDH
not related
WSU-DLCL2

E

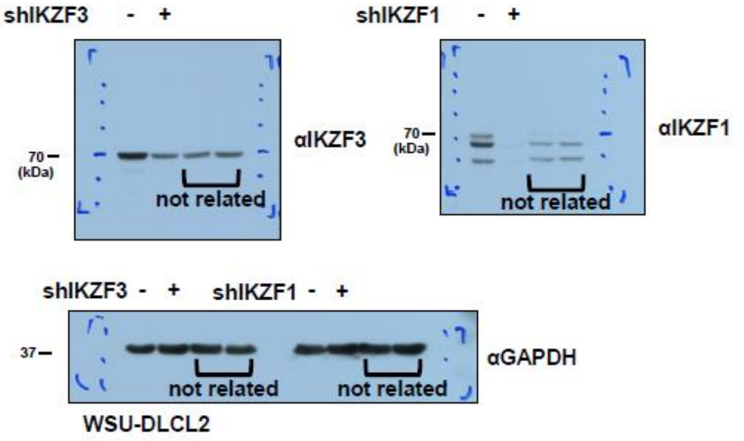

shIKZF3 - +
shIKZF1 - +
70 (kDa)
αIKZF3
αIKZF1
not related
not related
shIKZF3 - + shIKZF1 - +
37
αGAPDH
not related
not related
WSU-DLCL2

F

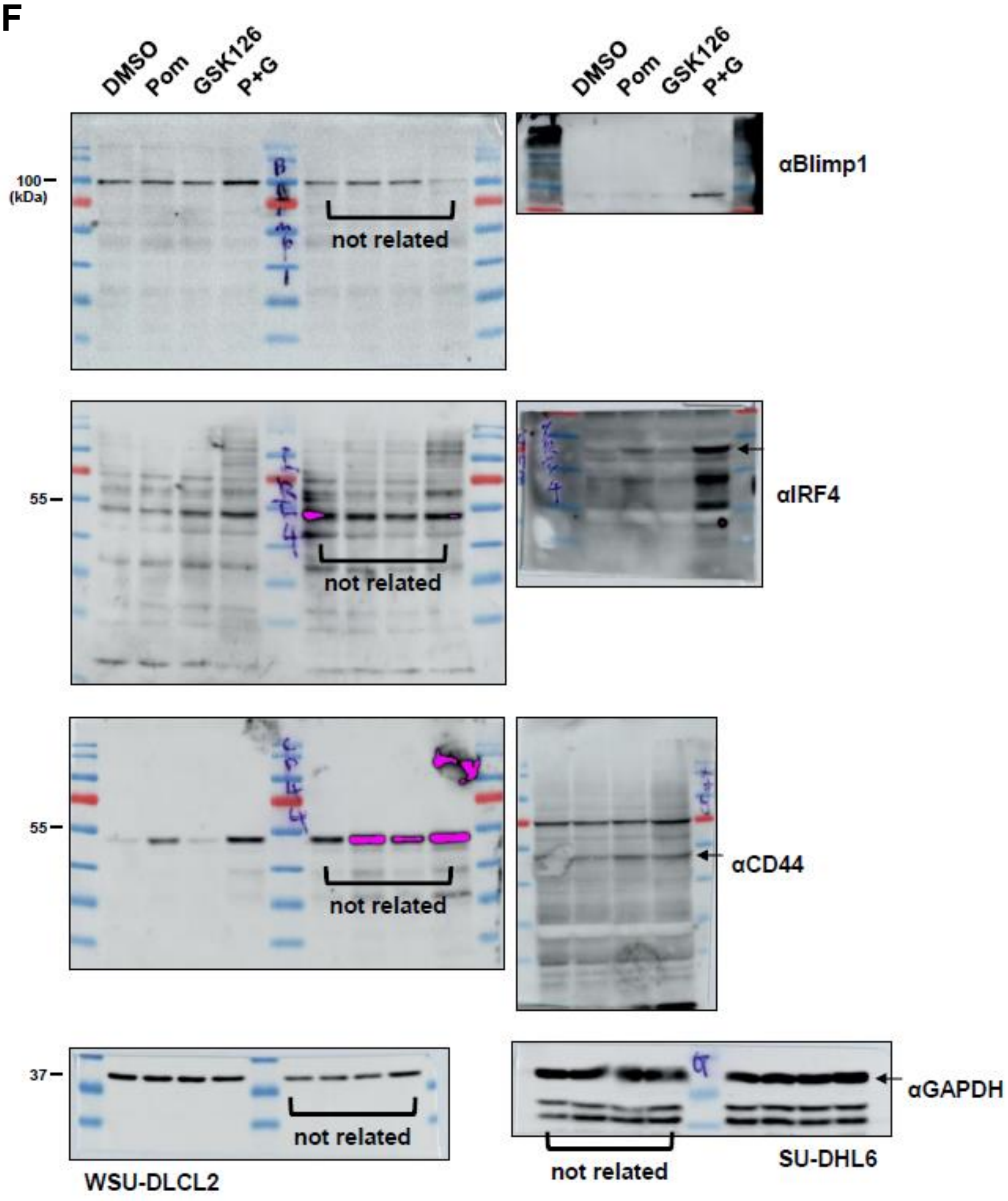

DMSO Pom GSK126 P+G
DMSO Pom GSK126 P+G
100 (kDa)
αBlimp1
not related
55
αIRF4
not related
55
αCD44
not related
37
αGAPDH
not related
not related
WSU-DLCL2
SU-DHL6

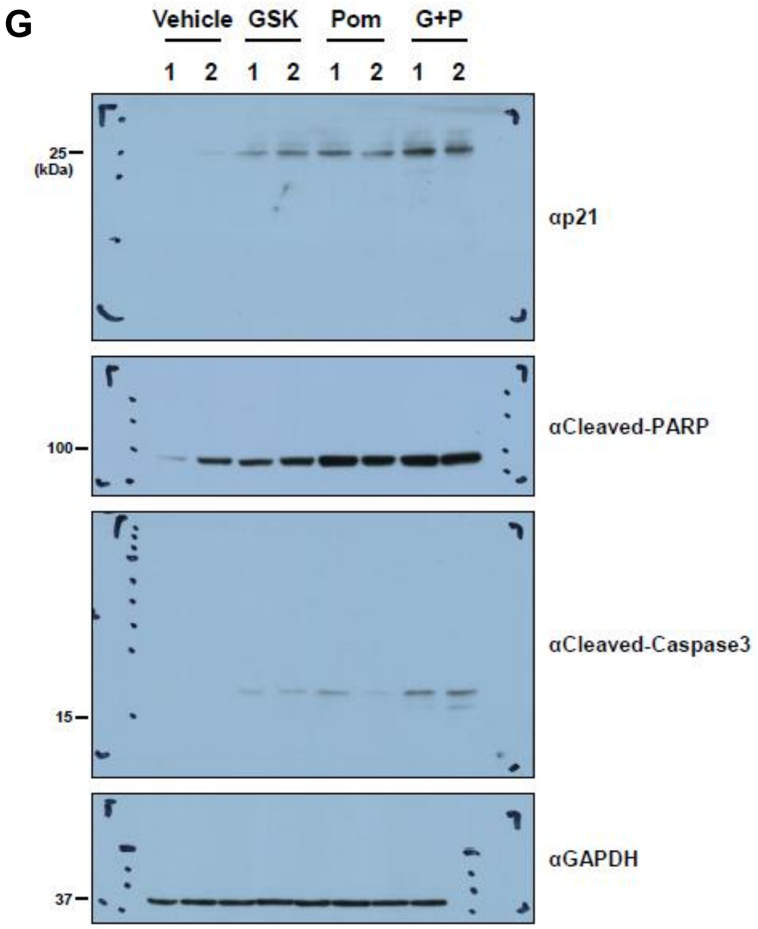

Figure S6. Original images of western blot. Original HRP-conjugated antibody images of (**A**) Figure 1E, (**B**) Figure 2E, (**C**) Figure 2H, (**D**) Figure 3A, (**E**) Figure 3D, (**F**) Figure 4J, (**G**) Supplementary figure 5B.

Table S1. Antibodies used for detect proteins.

Cat No.	Antibody	Company	RRID
ab176842	Histone H3	abcam	AB_2493104
ab6002	H3K27me3	abcam	AB_305237
ab198287	PRDM1/Blimp1	abcam	none
ab133590	MUM1/IRF4	abcam	none
ab157107	CD44	abcam	none
ab16667	Ki-67	abcam	AB_302459
#5246s	EZH2	CST	AB_10694683
#3737S	SUZ12	CST	AB_2196850
#14859S	IKZF1 (Ikaros)	CST	AB_2744523
#15103	IKZF3 (Aiolos)	CST	AB_2744524
#2947S	p21	CST	AB_823586
#9664S	cleaved-caspase3	CST	AB_2070042
#5625S	cleaved-PARP	CST	AB_10699459
#7074S	anti-Rabbit IgG HRP conjugated	CST	AB_2099233
HPA045910	CRBN	Sigma aldrich	AB_10960409
A9044	anti-Mouse IgG HRP conjugated	Sigma aldrich	AB_258431
T5168	αTubulin	Sigma aldrich	AB_477579
16818-1-AP	EED	Proteintech	AB_2262065
sc-47724	GAPDH	Santa Cruz	AB_627678

Table S2. shRNA targeting sequences for knockdown cell lines.

Gene	Target Seqeunce (5′ - 3′)
CRBN	GGGCTTGCAACTTGAATCTGATACTCGAGTATCAGATTCAAGTTGCAAGCTTTTTG
IKZF1	CCGGCCGTTGGTAAACCTCACAAATCTCGAGATTTGTGAGGTTTACCAACGGTTTTTG
IKZF3	CCGGGCTCTCGTACTGGACAGATTACTCGAGTAATCTGTCCAGTACGAGAGCTTTTT
EZH2 #1	CCGGGCTAGGTTAATTGGGACCAAACTCGAGTTTGGTCCCAATTAACCTAGCTTTTTG
EZH2 #2	CCGGCCCAACATAGATGGACCAAATCTCGAGATTTGGTCCATCTATGTTGGGTTTTTG

Table S3. Primer seqeunces for RT-qPCR.

Gene	Fwd (5′ - 3′)	Rev (5′ - 3′)
IL7	CTCCCCTGATCCTTGTTCTG	TCATTATTCAGGCAATTGCTACC
CD33	GAACACCCCCGATCTTCTCC	TGAGCACCGAGGAGTGAGTA
ITGA1	GGTGCTTATTGGTTCTCCGTTAG	TTCTCCTTTACTTCTGTGACATTGG
CSF1	ATGACAGACAGGTGGAACTGCCAG	TCACACAACTTCAGTAGGTTCAGG
CD44	CACGTGGAATACACCTGCAA	GACAAGTTTTGGTGGCACG
IRF4	AAAGGAAAGTTCCGAGAAGG	CGAAGGGTAAGGCGTTGT
Blimp-1	TCGGGTCGTTTACCCCATC	CACAGCGCTCAGGCCATTA
XBP1	TTACGAGAGAAAACTCATGGCC	GGGTCCAAGTTGTCCAGAATGC
Bcl6	CATGCAGAGATGTGCCTCCACA	TCAGAGAAGCGGCAGTCACACT
CD10	GGGGAGGCTTTATGTGGAAG	CTCGGATCTGTGCAATCAAA
SPIB	GGAGTGCTGCCCTGCCATAA	CCCCCACCCCAGATGAGATT
OBF	AGGTGAGGAGGATGTGATGACG	CGAATGCTTCTTGTCGTGACA
DYPD	GTTCTGGCTACCAGGCTAT	CATAAGGTGTTGTCCTGGAA
H1F0	CTGGCTGCCACGCCCAAGAA	CGGCCCTCTTGGCACTGGAC
RhoB	CATTCTGACCACACTTGTACGC	GGTTTCTTTTCCCTCTCCTTGT
SATB	CCTGGATTAGCCCTTTGG	CTGTGTGGTGGAACATTATG
CASP4	TTCCTGGCAATTGAAAATGG	TGCAAGCTGTACTAATGAAGGTG
ZNF98	AGGAGTGGCAATGCCTGGACAC	TCCTTGCTCCAGACAGGTGATC
HRPT1	GACCAGTCAACAGGGGACAT	AACACTTCGTGGGGTCCTTTTC
